# Wingless/Wnt Signaling in Intestinal Development, Homeostasis, Regeneration and Tumorigenesis: A Drosophila Perspective

**DOI:** 10.3390/jdb6020008

**Published:** 2018-03-28

**Authors:** Ai Tian, Hassina Benchabane, Yashi Ahmed

**Affiliations:** Department of Molecular and Systems Biology and the Norris Cotton Cancer Center, Geisel School of Medicine at Dartmouth College, Hanover, NH 03755, USA; ai.tian@dartmouth.edu (A.T.); hassina.benchabane@dartmouth.edu (H.B.)

**Keywords:** Wnt/Wingless signaling, Drosophila gut, animal model, intestinal physiology and pathology, Adenomatous polyposis coli (APC), colorectal cancer

## Abstract

In mammals, the Wnt/β-catenin signal transduction pathway regulates intestinal stem cell maintenance and proliferation, whereas Wnt pathway hyperactivation, resulting primarily from the inactivation of the tumor suppressor Adenomatous polyposis coli (APC), triggers the development of the vast majority of colorectal cancers. The Drosophila adult gut has recently emerged as a powerful model to elucidate the mechanisms by which Wingless/Wnt signaling regulates intestinal development, homeostasis, regeneration, and tumorigenesis. Herein, we review recent insights on the roles of Wnt signaling in Drosophila intestinal physiology and pathology.

## 1. The Canonical Wnt/β-Catenin Signaling Pathway

### 1.1. Wnt/β-Catenin Signaling Pathway

The canonical Wnt signaling pathway regulates the cytoplasmic level of the transcriptional coactivator β-catenin [1,2,3]. In the absence of Wnt ligand stimulation, cytoplasmic β-catenin is targeted for proteolysis by a “destruction complex”, which includes the two tumor suppressors Axin and Adenomatous polyposis coli (APC), and two kinases, glycogen synthase kinase 3 (GSK3) and casein kinase 1α (CK1α). The destruction complex promotes β-catenin phosphorylation, ubiquitination, and proteasomal degradation, thereby preventing the transcriptional regulation of Wnt target genes [4]. Binding of Wnt ligands to their co-receptors Frizzled (Fz) and low-density lipoprotein receptor-related protein 5/6 (LRP5/6; herein LRP6) activates signaling [5,6,7]. A consequent cascade of events assembles the “signalosome”, including the formation of a Fz-LRP6 complex, recruitment of Dishevelled (Dvl) to this complex, phosphorylation of the cytoplasmic tail of LRP6, and its association with Axin and GSK3 [8]. These events either result in the disassembly of the destruction complex, or in an alternative model, inhibit β-catenin ubiquitination within an intact complex [1,3,9,10]. In both of the models, β-catenin accumulates in the cytoplasm and translocates to the nucleus, resulting in its association with TCF and other transcriptional coactivators to regulate Wnt target gene expression [11,12,13].

### 1.2. Wnt/β-Catenin Signaling in Development and Disease

Wnt/β-catenin signaling regulates many cell behaviors in metazoans [2,14], including axis formation during development [15,16], maintenance of stem cell-replenished organs during adulthood [17,18,19], and faithful pattern restoration during tissue regeneration [17,20,21].

Because of the key roles of the Wnt pathway during development and homeostasis, its deregulation is associated with numerous congenital diseases, metabolic disorders, and cancers [2,22,23,24]. Notably, hyperactivation of Wnt signaling drives both the onset and the continued proliferation of colorectal cancer, which is among the leading causes of cancer-related death worldwide [18,25,26,27,28]. This underlies the intense effort to understand the roles of Wnt signaling in both intestinal physiology and pathology.

## 2. Wnt/β-Catenin Signaling in Mammalian Intestinal Physiology and Pathology 

A single layer of epithelial cells lines the lumen of the mammalian small intestine and colon, forming invaginations, termed crypts. The small intestinal epithelium also contains fingerlike protrusions termed villi. A massive renewal process, which is driven by intestinal stem cells (ISCs), replenishes the loss of differentiated intestinal epithelial cells [29]. Located at the crypt base, ISCs self-renew and give rise to transit-amplifying (TA) cells; the latter proliferate rapidly, migrate upwards, and differentiate into mature cells in the villi, where digestion and absorption are fulfilled [18,25,26]. Wnt pathway activity is graded at this site, with the highest levels at the base of the crypt [30,31,32,33]. Inhibition of Wnt signaling results in both abrupt cessation of proliferation and loss of ISCs, consequently leading to ablation of the intestinal epithelium [34,35,36,37,38,39]. Conversely, potentiation of Wnt signaling increases ISC number [40,41]. Together, these lines of evidence reveal the crucial roles of Wnt signaling in ISC self-renewal and proliferation during homeostasis. 

The aberrant activation of the Wnt pathway in ISCs promotes adenoma formation. Mutations in *APC* trigger this tumor-initiating step, underlying both the hereditary cancer syndrome, termed familial adenomatous polyposis (FAP) and the majority of sporadic cases (approximately 85%) of colorectal cancer [42,43,44,45,46]. Mutations affecting other components of the Wnt pathway substitute for *APC* mutations in most other colorectal cancer cases [42,46,47,48,49,50,51,52,53,54,55]. The subsequent acquisition of additional mutations in other pathways facilitates the progression of these adenomas to malignancy [56,57,58,59,60]. Notably, the restoration of APC in APC-deficient colorectal tumors triggers cell differentiation and re-establishes intestinal homeostasis [61]; thus, even late-stage tumors continue to rely on hyperactivated Wnt signaling to sustain their growth. This crucial requirement of Wnt pathway hyperactivation for both the initiation and the ongoing proliferation of colon cancer cells provides a potentially powerful target for therapeutic intervention. In recent years, several promising agents, including antibodies, small molecule inhibitors, and tailored peptides that interfere with Wnt pathway activation have been developed [2,53,62,63,64,65,66,67]. In particular, small molecule inhibitors of the ADP-ribose polymerase Tankyrase stabilize Axin and inhibit Wnt signaling in APC-deficient tumor cells and *Apc* mutant mice [68,69,70,71,72,73]. These observations highlight the great potential of drugging the Wnt pathway for treatment of colorectal cancers. However, as the Wnt pathway is required both in colon cancers and in normal stem cells, challenges remain in concomitantly achieving efficacy and safety [2,23,62,74]. A better understanding of the mechanistic differences that exist between physiological levels of Wnt signaling in the normal intestinal homeostasis versus the aberrantly increased levels found in pathologic states may provide selectivity between tumor and normal tissues, and is thus critical.

## 3. The Drosophila Adult Gut: A Powerful Model for Studying Wnt Signaling

**Figure 1:**
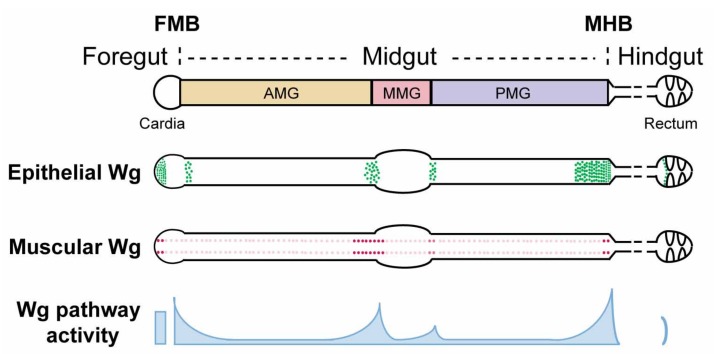
Schematic view of *wg* expression and Wg pathway activation in the Drosophila adult gut. The Drosophila adult gut is divided into foregut, midgut, and hindgut. The foregut/midgut boundary (FMB) and midgut/hindgut boundary (MHB) provide local niches for region-specific stem cells and contain critical valves that regulate food entry and exit. The midgut is further partitioned into anterior midgut (AMG), middle midgut (MMG), and posterior midgut (PMG), based on major constrictions and the existence of a specific acid-secreting region in the MMG. A *wg-gal4* knock-in line driving *UAS-lacZ* reveals *wg* expression in both the epithelium and the surrounding visceral muscle. At major compartment boundaries of the midgut, epithelial sources of *wg* are detected within enterocytes. In addition, four rows of *wg*-expressing cells are detected in the surrounding circular visceral muscles throughout the entire length of the midgut. Instead of being uniform, these muscle sources of *wg* are enriched at major compartment boundaries. Similarly, Wg pathway activation exists in gradients, exhibiting high-level expression at compartment boundaries and low-level expression throughout compartments.

Akin to the functional segmentation of the mammalian gastrointestinal tract [75,76,77,78,79,80], the Drosophila gut is subdivided into foregut, midgut, and hindgut, based on their distinct developmental origin and function. The midgut is further partitioned into compartments, termed the anterior, middle, and posterior midgut, with distinct digestive and metabolic functions, enterocyte architecture, gene expression profiles, and tumor susceptibility (Figure 1) [75,81,82,83,84,85,86]. Similar to that in its mammalian counterpart, Drosophila gut compartmentalization facilitates the sequential digestion of food and absorption of nutrients, as well as defense against infection. Resembling the mammalian digestive tract, the Drosophila adult midgut is comprised of a monolayer epithelium that is replenished regularly by ISCs [85,87,88]. ISCs give rise to either enteroblasts (EB) or pre-enteroendocrine cells (pre-EE), which subsequently differentiate into absorptive enterocytes (EC) or secretory enteroendocrine cells (EE), respectively [85,87,88,89,90,91]. 

Drosophila and mammalian guts share not only similar morphology, but also a requirement for Wnt signaling. One key difference is the reduced functional redundancy present in Wnt pathway components in Drosophila, providing a key advantage for elucidating their in vivo roles [15,92,93,94]. Furthermore, the ability to mark and manipulate stem cell lineages, to abrogate or to overactivate Wg signaling at defined time points, to study epithelial regeneration following injury, and to examine intestinal epithelial cell division, differentiation, and niche-stem cell contacts at the single cell level all add to the advantages of using Drosophila to study Wnt-driven physiology and pathology [91,95,96,97,98,99]. Moreover, the Drosophila gut also provides a powerful physiological context to test both novel Wnt pathway components and novel therapeutic agents that target the pathway [100,101,102,103]. 

## 4. Wg Signaling in the Drosophila Gut: Development, Homeostasis, Regeneration, and Tumorigenesis

### 4.1. Wg Is Expressed at Major Compartmental Boundaries in the Adult Midgut

The *wg* mRNA expression pattern in the adult gut has been determined using both in situ hybridization [104] and transcriptional reporters, including *wg-lacZ* (insertions of *lacZ* in the endogenous *wg* locus) [104,105,106,107,108], *wg-gal4* [109,110], *wg{KO, cherry}* (*cherry* knock-in at the endogenous *wg* locus) [106,111], and *wg{KO, gal4}* (*gal4* knock-in at the endogenous *wg* locus) [106,111,112]. These combined efforts revealed that Wg originates from both the gut epithelium and the visceral muscle. First, the level of *wg* expression in visceral muscles that surround the gut epithelium peaks at major intestinal compartment boundaries, including the foregut/midgut boundary (FMB), anterior/middle midgut boundary, middle/posterior midgut boundary, and midgut/hindgut boundary (MHB) [104,106] (Figure 1). Overexpression of *wg* in visceral muscle induces Wg target gene expression in the gut epithelium, suggesting that Wg derived from intestinal muscle communicates with the juxtaposed gut epithelium and instructs its behavior [106]. Second, *wg* is also expressed in the intestinal epithelium at major compartment boundaries including the FMB, anterior/middle midgut border, middle/posterior midgut border, MHB, and ileum/rectum border [106,107,109,110,112] (Figure 1). Specifically within midgut, *wg* expression is detected in enterocytes. Approximately 16 contiguous rows of cells in the adult terminal posterior midgut express *wg*, which is a significantly longer range than that present in the third instar larval wing imaginal disc. Whether there exist epithelial sources of Wg ligands inside the compartments (away from the boundaries) awaits further investigation. In summary, *wg* mRNA expression is enriched at compartment boundaries in both the gut epithelium and its overlying visceral muscle.

Studies with a monoclonal Wg antibody confirmed some of the expression pattern that was revealed by *wg* transcriptional reporters and in situ hybridization [104,110,112,113,114]. Wg protein is present in visceral muscles and is reduced upon RNAi-mediated knock down of *wg* specifically in muscle [104,113]. Furthermore, secreted Wg protein associates with progenitor cells within compartments during homeostasis, [104,112,113], and Wg protein levels are greatly increased during regeneration following injury [113] or upon the overexpression of the Ret receptor tyrosine kinase [112]. In addition, epithelial Wg protein is also detected at the MHB [110,114]. The presence of Wg protein at other intestinal compartment boundaries, and the source of the progenitor cell-associated Wg protein await further investigation, requiring reagents that permit an increased sensitivity in Wg protein detection. 

**Figure 2:**
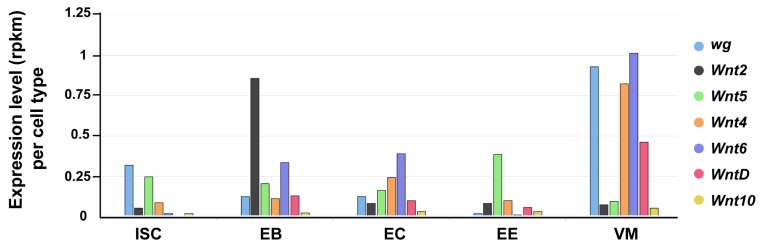
Expression of Drosophila Wnts in the gut. The seven Drosophila Wnt genes exhibit differential expression levels across distinct gut cell types (FlyGut-seq). ISC (intestinal stem cell); EB (enteroblast); EC (enterocyte); EE (enteroendocrine cell); and, VM (visceral muscle).

The Drosophila genome encodes seven Wnt genes [115]. In addition to Wg, the other six Wnts (Wnt2, Wnt4, Wnt5, Wnt6, Wnt10, and WntD) are also expressed in the Drosophila intestine (FlyGut-seq [98]; Figure 2). Their precise expression pattern and contribution to the intestinal physiology awaits further work.

### 4.2. Graded Activation of Wg Signaling at Major Compartment Boundaries in the Drosophila Midgut

The distinct regions in the Drosophila adult intestine at which Wg signaling is active have been identified by the analysis of Wg pathway target genes [81,95,106]. *frizzled 3* (*fz3)* and *naked cuticle* (*nkd)* are target genes that are activated directly by Armadillo/β-catenin-TCF in several physiological contexts and are feedback antagonists of the Wg pathway [116,117,118]. *Fz3-RFP*, which is a 2.3 kb promoter fusion line [119], and *nkd-lacZ*, an insertion of *lacZ* in the endogenous *nkd* locus [118], respond to both loss and gain of Wg signaling in distinct developmental contexts [118,119,120,121,122,123]. The specificity of these two target gene reporters in the intestine has been verified by mutant clonal analysis of essential Wg pathway components [101,106]. In the adult gut epithelium, *fz3-RFP* and *nkd-lacZ* exhibit overlapping graded expression patterns, peaking at major compartment boundaries and decreasing as a function of distance from the boundaries [81,101,106,107]. Asymmetric gradients of Wingless target genes are present at the AMG/MMG boundary and the MHB boundary [81,106]. In addition, low-level expression of Wg target genes is also present within the interior of compartments [81,101,106]. Thus, Wg pathway activation peaks at the boundaries between intestinal compartments, decreases with distance from the boundaries, and is the lowest in the interior of compartments.

Loss-of-function clonal analysis of essential Wg pathway components also revealed that Wg stimulation activates *fz3-RFP* and *nkd-lacZ* expression specifically in ECs, both at the intestinal compartment boundaries and within the interior of compartments [106]. Wg-dependent activation of *fz3-RFP* expression also occurs in progenitor cells, but only those in the posterior terminal midgut [106,107]. Thus, under physiological conditions, Wg signaling is transduced within enterocytes along the entire midgut, and also in progenitor cells in the posterior terminal midgut. By contrast, in *Apc1 Apc2* double null mutant clones, *fz3-RFP* expression is greatly induced in all intestinal cell types, including progenitor cells, EEs, and ECs [106]. Thus, all of the gut epithelial cells have the capacity to activate Wg signaling. The mechanism that restricts Wg pathway activation to a subset of intestinal epithelial cells under physiological conditions awaits further investigation, and may require improvement in the sensitivity of detection of destruction complex components in the Drosophila gut. 

### 4.3. Wg Directs Pattern Formation during Drosophila Gut Development

The major regions of the Drosophila intestine are derived from distinct germ layers: the midgut arises from endoderm, whereas both the foregut and hindgut arise from ectoderm [124,125]. The midgut epithelium is generated from adult midgut precursors (AMPs) that are initially specified during embryogenesis [126,127]. During larval stages, AMPs undergo proliferation in clustered islets and are encapsulated by their own differentiated daughters, termed peripheral cells (PCs) [126,127,128,129,130]. At the onset of metamorphosis, the larval midgut epithelium degenerates, leaving intact only regions that are near the foregut/midgut and midgut/hindgut borders [107,110,114,127,131,132]. The PCs, which serve as a transient niche that prevents AMP differentiation in the larval gut, degenerate at this stage. The released AMPs undergo rapid proliferation, differentiation and dispersal, and the resulting gut primordia merge and elongate to rebuild the adult midgut epithelium. During this process, the vast majority of AMPs differentiate into ECs or EEs, whereas small subsets become the future adult ISCs [127,128,129,130,133,134,135,136]. Concomitantly, the surrounding visceral muscles contract and remodel, undergoing dedifferentiation and redifferentiation [137,138]. In contrast to the midgut, the developmental processes that rebuild the adult foregut and hindgut remain under debate. In one model, progenitors proliferate in defined zones at the foregut/midgut and midgut/hindgut borders, differentiate and extend cephalically or caudally during metamorphosis to replace the larval epithelium, and thereby give rise to the adult foregut and hindgut [110]. In an alternative model for hindgut development, the adult pylorus, ileum, and rectum are derived from independent larval precursors [114]. 

The complex developmental processes that direct formation of the Drosophila gut require precise spatiotemporal orchestration. How is this achieved? Whether Wnt/Wg signaling provides instructive signals for the development of distinct gut compartments, and the boundaries that separate them has been recently investigated. These studies shed light on three zones that are enriched for Wg pathway activation: the two distal boundaries that separate midgut from foregut and hindgut, and the copper cell region in the middle midgut.

#### 4.3.1. Wg Signaling in Formation of the Adult Intestinal Midgut/Hindgut Boundary during Pupation

The midgut/hindgut boundary (MHB) partitions the endoderm-derived posterior terminal midgut from the ectoderm-derived anterior hindgut. Due to their distinct developmental origins, midgut and hindgut cells at this boundary exhibit distinct characteristics with respect to cell size, nuclear size, cell adhesion proteins, proliferation rate, presence of cuticle versus microvilli-rich brush border, and the overlying visceral muscle [106,107,110,114,132]. Juxtaposed posteriorly with the MHB, the anteriormost adult hindgut cells, also termed the hindgut proliferation zone (HPZ), are formed by expansion of the hindgut progenitor cells during pupation [110]. The precise mechanism underlying the formation of the posterior terminal midgut, which lies immediately anterior to the MHB, remains uncertain. In one model, the posterior terminal midgut is formed by bidirectional movement of neighboring cell populations and their subsequent adoption of new cell fates during metamorphosis [132]. Specifically, the anteriormost larval hindgut cells cross the MHB, lose their hindgut identity and subsequently transdifferentiate into posterior terminal midgut enterocytes. Concurrently, AMPs that are located immediately anterior to the posterior terminal midgut migrate posteriorly and give rise to the posterior terminal midgut progenitor cells and EEs [132]. In an alternative model, rather than undergoing transdifferentiation, a hybrid progenitor cell acts early in larval development to produce the posterior terminal midgut, MHB and pylorus [107]. 

Remarkably, Wg is secreted from three distinct sources at the MHB: epithelial cells in the posterior terminal midgut, a ring of epithelial cells at the anteriormost hindgut, and muscle fibers overlying the posterior terminal midgut [106,107,110,114,132] (Figure 1). Together, these three sources contribute to the high levels of secreted Wg that induce high level Wg pathway activation around the MHB [81,106,107], which is crucial for the proper development of this region in at least three distinct aspects. First, the level of Wg activity specifies the size of the anteriormost hindgut region (HPZ); Wg pathway hyperactivation induces HPZ expansion, whereas Wg signaling inhibition leads to adult hindgut loss [110]. Similarly, Wg pathway activation also determines the size of the posterior terminal midgut as Wg overexpression results in an abnormally enlarged posterior terminal midgut [132]. Whether this defect results from an aberrantly increased number of hindgut progenitor cells that migrate anteriorly or from an expanded proportion of the hybrid progenitor population that adopts terminal midgut cell fate remains unclear. Third, Wg signaling provides positional cues for cells to adopt proper cell fate in the reformation of the MHB region during pupation and to prevent lineage mixing, the inhibition of which leads to two additional phenotypes [106]. In the first phenotypic class, Wg signaling-defective cells in the posterior terminal midgut fail to adopt a midgut fate and exhibit characteristics of hindgut epithelia (small nuclear and cell size, and expression of hindgut-specific markers) and are “tightly-packed” in a spiral pattern. Consequently, they segregate from the neighboring wild-type midgut epithelium as discrete domains. It is thus possible that Wg signaling instructs the transdifferentiation of hindgut cells to midgut cells following their migration into the midgut, or alternatively, graded Wg pathway activation might be required to specify individual cell fates within the population of hybrid progenitors during metamorphosis. In the second phenotypic class, Wg signaling-defective posterior terminal midgut cells display abnormally large nuclear and cell size. These cells most likely derive from posteriorly-migrating AMPs that fail to adopt posterior terminal midgut cell fate following their migration. Furthermore, in contrast to their normal restriction within the posterior terminal midgut, some of these abnormally large cells invade the hindgut. Together, these recent observations suggest that Wg signaling is critical for cell sorting, patterning, and lineage separation during the reformation of the MHB during metamorphosis. 

#### 4.3.2. Wg Signaling in Formation of the Foregut/Midgut Boundary of the Adult Gut during Development

High levels of Wg protein and Wg pathway activity are present not only at the MHB, but also at the foregut/midgut boundary (FMB) [106,109]. Disruption of Wingless signaling during development gives rise to cells of abnormal size and alignment at this boundary, distorting the normal structure of the cardia [106]. Thus, as is the case for the MHB, Wg signaling also instructs the proper cell fate specification at the FMB during development. 

#### 4.3.3. Wg Signaling in Embryonic and Larval Gut Development 

Wg signaling is also required during embryonic and larval gut development. During embryogenesis, Wg signaling directs left-right asymmetry of the foregut and the anterior midgut, disruption of which results in left-right inversion or loss of laterality [139]. In addition, Wg-dependent patterning is essential for the development of the embryonic hindgut and rectum [140]. In the larval midgut, high levels of Wg and Wg pathway activation are present at the boundaries of the middle midgut region [106,141], which contains two distinct cell populations: the anterior acid-secreting “copper cells” and the posterior “large flat cells”. Nearly all of the presumptive middle midgut cells have the intrinsic capacity to adopt either cell fate during development [141]. Two different thresholds of Wg concentration specify their fates: low levels of Wg promote copper cell fate, whereas high levels of Wg repress copper cell fate and promote the large flat cell fate [141]. Whether Wg signaling plays a similar role during the development of the adult middle midgut awaits further investigation. In summary, Wg is crucial to instruct proper patterning of the gut throughout development.

### 4.4. Wg Signaling Regulates ISC Self-Renewal/Maintenance and Proliferation in the Drosophila Adult Gut during Homeostasis 

When nutrients are plentiful, the intestinal epithelium is replenished in a highly regulated process. Several findings support a role for Wg signaling in ISC self-renewal/maintenance. In distinct compartments along the anterior-posterior axis of the Drosophila gut, dominant negative TCF results in rapid loss of ISC lineages in the posterior midgut [104,113,142], the CCR ([143], and the cardia [109]. In the posterior midgut, *fz fz2* double mutant cells, as well as *arm*, or *dsh* mutant cells are lost over time [104]. In addition, ISC number is reduced by the temperature sensitive *wg* mutant allele (*wg^ts^*) [104] and increased upon Wg overexpression [104,109,144]. However, controversy remains regarding the requirement for Wg signaling in ISC self-renewal, as: (1) ISC self-renewal is not affected upon concomitant inactivation of *Apc1* and *Apc2* in the posterior midgut [142]; (2) concomitant knockdown of *wg* from epithelial and muscle sources or in *wg^CX4^* heterozygous mutants does not lead to significant loss of posterior midgut ISCs even after 30 days [113]; and, (3) in contrast with the effects of dominant negative TCF overexpression, inactivation of core Wnt pathway components with null alleles results only in mild effects on ISC maintenance during homeostasis [104]. Thus, dominant negative TCF may cause non-specific effects that muddle the role of Wg signaling in ISC self-renewal. In addition, contradictory observations have also left the role of Wingless signaling on ISC proliferation uncertain [104,113].

More recently, several studies have demonstrated that Wg signaling is critical for intestinal homeostasis, but active primarily in enterocytes rather than in ISCs [81,101,106]. Wg signaling in enterocytes non-autonomously regulates JAK-STAT signaling in neighboring ISCs, thereby preventing ISC overproliferation during homeostasis. Together, these recent studies reveal that Wg signaling is essential to prevent aberrant increases in ISC proliferation during homeostasis. 

### 4.5. Wg Signaling in Adult Midgut and Hindgut Regeneration Following Injury

During adulthood, the Drosophila intestinal epithelium may be exposed to damage from bacterial infection, chemical toxins, or mechanical stress. To repair the resultant injury, mechanisms have evolved to regenerate the damaged intestinal epithelium. Intestinal cells sense damage, induce compensatory ISC proliferation and differentiation to replenish the lost epithelium, and subsequently re-establish homeostasis [145,146,147]. This rapid and effective regeneration process depends on multiple signaling pathways, including Wg.

Following exposure to cytotoxic agents or bacterial infection, the level of Wg protein increases markedly in EBs of the midgut epithelium and induces compensatory proliferation of ISCs, whereas *wg* knockdown strongly impairs this response [113]. Similarly, the abrogation of Wg signaling in the intestinal epithelium abolishes gut regeneration [113]. Thus, upregulation of Wg levels and activation of the Wg pathway in the intestinal epithelium are essential for damage-induced regeneration of the Drosophila midgut. 

In contrast to the midgut, the Drosophila adult hindgut lacks active stem cells, and, following damage, preserves epithelial integrity in part by endoreplication and cellular hypertrophy in the pylorus [107,114,148]. In addition, a unique population of ISCs in the posterior terminal midgut that are normally quiescent proliferate robustly following injury in the Wg-enriched MHB or the hindgut [107]. As noted above, epithelial Wg ligand is detected in the region of the MHB [106,107]. These Wg positive cells express both midgut and hindgut markers, and thus constitute a hybrid zone (HZ) between the midgut and hindgut [107]. In contrast to other ISCs in the midgut, ISCs that are located immediately anterior to the HZ are responsive to Wg stimulation even under homeostatic conditions [106,107] and have been termed organ boundary intestinal stem cells (OB-ISCs) [107]. Damage in the HZ and pylorus upregulates expression of the cytokine *upd3* in the Wg-enriched HZ. In response, the OB-ISCs undergo both symmetric and asymmetric divisions to give rise to new OB-ISCs and enterocytes. When the injury of the HZ is severe, hyperplastic midgut OB-ISCs cross the MHB through gaps in the injured HZ [107]. Thus, the OB-ISCs are a unique population of Wg-responsive midgut ISCs that display robust cell proliferation in response to cell loss in the HZ and pylorus; however, whether their compensatory response requires Wg signaling awaits further investigation. 

### 4.6. Hyperactivation of Wg Signaling Due to Loss of Apc: Initiation and Progression of Tumorigenesis in the Drosophila Gut 

#### 4.6.1. Initiation of Intestinal Tumorigenesis upon Loss of Apc 

The Drosophila melanogaster genome encodes two *Apc* genes: *Apc1* and *Apc2* [149,150,151,152,153,154]. Either concomitant inactivation of both Drosophila *Apc* homologs, or inactivation of *Apc1* singly, leads to overproliferation of ISCs, epithelial hyperplasia, and disrupted epithelial cell polarity, resulting in the formation of a multilayered epithelium [100,142,144,155,156,157,158]. These defects resemble mammalian intestinal adenomas that arise following loss of APC, highlighting the potential of using the Drosophila *Apc1* mutant as a model to study colorectal cancers. In *Apc1* mutants, ISC overproliferation begins during pupation, whereas the disruption of epithelial cell polarity occurs after eclosion [100]. Thus, the defects caused by loss of Apc1 begin during development and increase in severity during adulthood. These defects are mediated by the hyperactivation of Wg signaling, as knockdown of the transcriptional co-activator Pygopus, expression of dominant negative TCF, or the inactivation of two transcriptional regulators of the Wg pathway, Earthbound (Ebd) and Erect wing (Ewg), suppress the *Apc1* mutant phenotype [100,142,157]. 

Despite known roles for Apc2 in the mammalian intestine [159,160], the role of Drosophila Apc2 and whether there exists some redundancy between Apc1 and Apc2 in the Drosophila midgut remains uncertain. One report suggested that functional redundancy exists between the two proteins, as inactivation of both *Apc1* and *Apc2* was required for ISC overproliferation, multilayering of the epithelium, and the upregulation of a Wg target gene reporter [157]. However, several other studies have revealed that inactivation of *Apc1* singly is sufficient to fully account for these effects of Wg pathway hyperactivation [100,144,155]. 

#### 4.6.2. Progression of Intestinal Tumorigenesis Following Apc Loss 

As noted above, in mammals, the acquisition of additional mutations transforms pre-malignant adenomas to malignant carcinoma following the loss of APC [56,57,58,59,60]. Recent studies have recapitulated this tumor progression process in the Drosophila digestive tract and shed light on three underlying molecular mechanisms. 

First, cell competition exists between Drosophila *Apc1 Apc2* double mutant tumor cells and adjacent wild-type cells [156]. Epithelial cells bearing *Apc* mutations act as “super competitors”, trigger apoptosis in the surrounding wild-type cells, clear space for dissemination, and result in host tissue attrition. Remarkably, inhibition of cell competition by the blocking of apoptosis prevents *Apc* mutant tumor expansion [156]. Thus, host-tumor cell competition is essential for tumor growth in *Apc* mutant midguts. 

Second, two independent studies examined the effects of hyperactivated Ras in *Apc* mutant midgut cells (*Apc1 Apc2-Ras^V12^* clones) [157,158]; both indicated that the oncogenic activation of Ras exacerbates the phenotypes caused by *Apc* loss alone. Specifically, *Apc-Ras^V12^* double mutant clones exhibit hallmarks of malignant transformation that include the inhibition of cell differentiation, disruption of cell polarity, and invasive outgrowth. As a result, intestinal physiology deteriorates with time and lifespan is reduced. Therefore, oncogenic Ras activation synergizes with *Apc* loss to promote intestinal tumor progression [157,158]. Local juvenile hormone activity derived from the gut progenitors is required for this process [161]. 

Third, genome sequencing data not only confirmed that in human colorectal cancers, multiple mutations are acquired in addition to *APC,* but also revealed that these mutations exist in distinct combinations in different tumors [42,46,103,162,163]. To examine the effect of genetic complexity and heterogeneity on gut tumor progression and drug response, a recent study took advantage of the powerful genetic tools that exist in Drosophila, generating 32 distinct multigenic (quadruples or quintuple) alterations that are based on patient tumor data [103]. The effects of these alterations were examined in the Drosophila hindgut, which revealed that the interaction between concurrent mutations promoted robust epithelial cell transformation. Moreover, drug resistance also emerged in these multigenic combinations. With the knowledge gained from these models, the order of drug treatment was manipulated to promote drug sensitivity in Drosophila tumor cells, and this approach was subsequently validated in mammalian models [103]. Together, these findings demonstrate that the Drosophila adult digestive tract recapitulates key events in both the initiation and progression of tumorigenesis following APC loss, and also offers a promising platform for both drug screening and the identification of novel tumor modifiers. 

## 5. The Drosophila Gut as a Powerful In Vivo Context to Test Novel Therapeutic Agents and Novel Wnt Pathway Components 

Our understanding of the roles of Wg signaling in Drosophila intestinal physiology and pathology has been greatly improved in recent years. These advances have, in return, prompted the use of the Drosophila gut as a powerful physiological context to examine both novel therapeutic agents and novel Wnt pathway components. Examples of such approaches targeting different levels of Wnt signaling are summarized below.

### 5.1. At the Receptor Level: The Signalosome

A recent study revealed, unexpectedly, that in *APC*-deficient colorectal carcinoma cells, blocking signalosome formation by knocking down *LRP6*, *Fz,* or *DVL* reduces β-catenin nuclear accumulation and inhibits constitutive Wnt pathway activation. Thus, signalosome assembly is essential for aberrantly increased Wnt signaling following loss of APC [164,165]. This hypothesis was further tested in the in vivo context of Drosophila *Apc1* mutant midguts. Notably, knocking down either *arr* or *dsh* rescues *Apc1* mutant intestinal defects, including ISC overproliferation, epithelial cell polarity disruption, and aberrant activation of Wg target genes [164]. Thus, there exists an evolutionarily conserved dependence on signalosome assembly for Wnt pathway hyperactivation following the loss of APC. This process requires clathrin-mediated endocytosis, but is independent of Wnt ligands [164]. 

### 5.2. In the Cytoplasm: Tankyrase

Tankyrase (Tnks) is an ADP-ribose polymerase that targets Axin for proteolysis [69,72]. Small molecule inhibitors of Tnks disrupt Wnt signaling in cultured human cells and reduce colonic adenoma growth in mouse models, suggesting that Tnks is a promising therapeutic candidate for the treatment of Wnt-driven cancers [68,69,70,71,72,73]. However, the physiological settings in which Tnks is required to promote Wnt signaling had been unclear [68,69,166]. One of the complications is functional redundancy in the two Tnks paralogs in vertebrates [167]. Drosophila genomes encode only one Tnks, which is highly conserved with its vertebrate homologs. Capitalizing on Drosophila genetics, null alleles of *Tnks* were generated. In conditions of limited nutrient supply, these *Tnks* mutant adults displayed markedly increased mortality, suggesting disrupted digestive function [101]. Examination of the adult intestines from *Tnks* null mutants revealed several physiological requirements [101]. First, Tnks is crucial to maintain Axin levels below a physiological threshold and this is essential for the control of ISC proliferation; *Tnks* mutants display severe ISC overproliferation. Second, Tnks is essential to ensure proper activation of Wg signaling in the midgut, which provided the first in vivo evidence that regulation of Axin by Tnks is required for Wg target gene activation in a physiological context. Notably, this requirement for Tnks in Wg pathway activation is spatially restricted: Tnks is essential for Wg signaling only in regions where Wg pathway activity is relatively low, but is dispensable where pathway activity is high, reflecting the role of Tnks in the amplification of Wg signaling in vivo. 

### 5.3. In the Nucleus: Earthbound and Erect Wing

Due to the requirements for Wnt signaling in both normal homeostasis and Wnt-driven cancers, one of the major challenges for the therapeutic targeting of this pathway is to concomitantly achieve efficacy and specificity [2,23,62,74]. The discovery of transcription cofactors that are essential for hyperactivated signaling but dispensable for physiological processes distinguished tumors from normal tissues [168,169,170,171,172,173,174,175,176]. Through a forward genetic screen in Drosophila, two novel suppressors of Apc1, Earthbound (Ebd) and Erect wing (Ewg), were identified as evolutionarily conserved transcription cofactors of the Wnt pathway that physically interact with each other and with Armadillo-Tcf [177,178]. Remarkably, both Ebd and Ewg are essential mediators of the pathological consequences of Apc1 inactivation in the intestine: aberrantly increased number of progenitors, defects in adhesion and epithelial polarity, disorganization of the intestinal architecture and widespread deregulation of Wg target gene expression. In contrast, during intestinal homeostasis, Ebd is required for the Wg-dependent control of ISC proliferation, whereas Ewg is dispensable [100]. Therefore, Ebd and Ewg are differentially required in physiological Wnt pathway activation versus oncogenic Wnt pathway hyperactivation following Apc1 loss, conferring mechanistic differences in the Wnt transcription machinery, and providing potential selectivity between normal tissues and tumors. In addition, these findings also provided in vivo evidence that the core β-catenin-TCF transcriptional machinery is insufficient for the transformation of intestinal epithelial cells in *Apc1* mutants; cooperation of β-catenin-TCF with Ebd and Ewg is also necessary. Further, these findings suggest that the human homolog of Ebd, Jerky (also known as JRK or JH8) [177,179,180,181,182,183,184], and the human homolog of Ewg, Nuclear respiratory Factor 1 (NRF1) [178,185,186,187], may provide promising drug targets for the treatment of Wnt-driven cancers. Notably, Jerky was identified in a high-throughput RNAi screen that facilitates Wnt target gene activation in colon adenocarcinoma cells [188]. Two later studies validated this role of Jerky as a positive modulator in Wnt signaling in colon cancer cell lines and further revealed that this is achieved by promoting the association of β-catenin and TCF and the recruitment of β-catenin to chromatin [177,189]. Moreover, aberrantly high levels of Jerky are present in human colorectal tumors [189]. A possible role for NRF1 in Wnt signaling awaits future investigation. 

## 6. Crosstalk between Wg Signaling and Other Signaling Pathways in the Drosophila Gut 

Several signaling pathways are involved in Drosophila intestinal physiology and pathology, thus weaving an intricate regulation network [190]. Recent studies have revealed the crosstalk between Wg signaling and other signaling pathways during homeostasis, regeneration, and tumorigenesis. 

First, during homeostasis, Wg signaling prevents the aberrant activation of the JAK-STAT pathway [106]. Specifically, diminishing Wg signaling results in a marked increase in the expression of the JAK-STAT pathway ligands Upd2 and Upd3 in the enterocytes, which in turn triggers the aberrant activation of JAK-STAT signaling in the neighboring ISCs and drives their non-autonomous overproliferation. 

Second, damage to the intestinal epithelium leads to the overactivation of the JNK pathway and at the same time also results in increased levels of the Wg ligand [113,191]. This upregulation of Wg is dependent on JNK pathway activation, but not vice versa, thus placing Wg signaling downstream of JNK pathway activation during the regeneration process [113]. 

Third, both the JAK-STAT pathway and the EGFR pathway are hyperactivated in the Drosophila gut upon loss of Apc1 [144]. Remarkably, disruption of either JAK-STAT signaling or EGFR signaling completely suppresses the intestinal hyperplasia resulting from Apc1 loss, revealing the underlying signaling networks at the tumor initiation step. 

## 7. Conclusions

The evolutionary conservation of Wnt/Wingless signaling and the similarities between the Drosophila and mammalian digestive tracts have made the Drosophila gut a powerful model to study intestinal physiology and pathology. Recent advances have uncovered critical roles for Wg signaling in development, homeostasis, and regeneration of the Drosophila adult gut. Furthermore, the Drosophila gut has become a model for colorectal tumorigenesis. As the Drosophila gut has proven to be an effective platform for drug screens [102,103], *Apc1* mutant guts could serve as a potential platform to identify novel compounds that combat Wnt-driven cancers. In addition, recent work has revealed the importance of the Drosophila gut model for elucidating context-specific functions of newly identified Wg pathway components. Lastly, the unique characteristics of the adult gut have been advantageous for tackling basic questions in cell biology, including cell-cell competition and interorgan communication. 

## 8. Future Perspectives

Many questions remain, including how Wg signaling gradients are established at the compartment boundaries, how they are maintained during the normal turnover of the intestinal epithelium, and how they recover following injury. In addition, whereas it is known that ISCs residing in distinct compartments along the anterior-posterior axis of the Drosophila gut have different identities and exhibit different proliferate rates [81,82], whether distinct Wg responses exist among these different ISC populations awaits investigation. Moreover, how Wg interfaces with other signaling pathways that are known to regulate development, homeostasis, and regeneration of the adult gut remains an open question. The powerful genetic techniques that have been developed to dissect the biology of the Drosophila gut will pave the way for future studies that address these questions, shedding more light on a pathway that is critical for development and disease.
